# Impact of COVID-19 Outbreak on Cancer Patient Care and Treatment: Data from an Outpatient Oncology Clinic in Lombardy (Italy)

**DOI:** 10.3390/cancers12102941

**Published:** 2020-10-12

**Authors:** Erica Quaquarini, Giuseppe Saltalamacchia, Daniele Presti, Giulia Caldana, Valentina Tibollo, Alberto Malovini, Raffaella Palumbo, Cristina Maria Teragni, Emanuela Balletti, Ludovica Mollica, Elisa Biscaldi, Mara Frascaroli, Antonio Bernardo, Federico Sottotetti

**Affiliations:** 1 Medical Oncology Unit, ICS Maugeri-IRCCS SpA SB, 27100 Pavia, Italy; giuseppe.saltalamacchia@icsmaugeri.it (G.S.); daniele.presti@icsmaugeri.it (D.P.); raffaella.palumbo@icsmaugeri.it (R.P.); cristina.teragni@icsmaugeri.it (C.M.T.); emanuela.balletti@icsmaugeri.it (E.B.); ludovica.mollica@icsmaugeri.it (L.M.); antonio.bernardo@icsmaugeri.it (A.B.); federico.sottotetti@icsmaugeri.it (F.S.); 2 Experimental Medicine School, University of Pavia, 27100 Pavia, Italy; 3 Translational Oncology Unit, ICS Maugeri-IRCCS SpA SB, 27100 Pavia, Italy; giulia.caldana@icsmaugeri.it (G.C.); elisa.biscaldi@icsmaugeri.it (E.B.); mara.frascaroli@icsmaugeri.it (M.F.); 4 Laboratory of Informatics and System Engineering for Clinical Research, ICS Maugeri-IRCCS SpA SB, 27100 Pavia, Italy; valentina.tibollo@icsmaugeri.it (V.T.); alberto.malovini@icsmaugeri.it (A.M.)

**Keywords:** cancer, containment measures, lockdown, SARS-CoV-2, systemic treatment

## Abstract

**Simple Summary:**

Cancer has been reported as a major risk factor for adverse outcomes and death during COVID-19 pandemic. The aim of this study was to investigate the management of cancer patients and oncological treatments during the COVID-19 pandemic and to describe the containment measures performed in ICS Maugeri outpatient clinic (Pavia, Italy). A comparison with the same period of the four previous years was performed. A significant reduction in access for therapy was seen between the year 2020 and 2019 but not between 2020 and 2018, 2017, or 2016. In 2020, the “pandemic fear” was the most common cause of treatment delay. Only few patients developed COVID-19. A significant reduction in radiological exams was found in 2020 versus all the other years considered. The low incidence of COVID-19 among our cancer patients, along with the hospital policy to control infection, enabled safe cancer treatment and a continuum of care in most patients.

**Abstract:**

Lombardy was the first area in Italy to have an outbreak of coronavirus disease 19 (COVID-19) at the beginning of 2020. In this context, cancer has been reported as a major risk factor for adverse outcomes and death, so oncology societies have quickly released guidelines on cancer care during the pandemic. The aim of this study was to investigate the management of cancer patients and oncological treatments during the COVID-19 pandemic and to describe the containment measures performed in our outpatient clinic at Pavia (Lombardy). A comparison with the same period of the four previous years (2019, 2018, 2017, and 2016) was also performed. Using our electronic databases, we evaluated the number and characteristics of patients accessing the hospital for anticancer drug infusion from 24 February, 2020 to 30 April, 2020 and the number of radiological exams performed. Although a significant reduction in access for therapy was seen when compared with 2019 (2590 versus 2974, access rate ratio (ARR) = 0.85, *p* < 0.001), no significant differences in access numbers and ARR was evident between 2020 and 2018, 2017, or 2016 (2590 versus 2626 (ARR = 0.07), 2660 (ARR = 0.99), and 2694 (ARR = 0.96), respectively, *p* > 0.05). In 2020, 63 patients delayed treatment: 38% for “pandemic fear”, 18% for travel restrictions, 13% for quarantine, 18% for flu syndrome other than COVID-19, and 13% for worsening of clinical conditions and death. Only 7/469 patients developed COVID-19. A significant reduction in radiological exams was found in 2020 versus all the other years considered (211 versus 360, 355, 385, 390 for the years 2020, 2019, 2018, 2017, and 2016, respectively, *p* < 0.001). The low incidence of COVID-19 among our cancer patients, along with the hospital policy to control infection, enabled safe cancer treatment and a continuum of care in most patients, while a small fraction of patients experienced a therapeutic delay due to patient-related reasons.

## 1. Introduction

Coronavirus disease 2019 (COVID-19) is a systemic infectious disease caused by severe acute respiratory syndrome coronavirus 2 (SARS-CoV-2), which was first recognized in China at the end of December 2019, later becoming a pandemic [1].

On 20 February, 2020, a severe case of pneumonia due to SARS-CoV-2 was diagnosed in Northern Italy, in the Lombardy region [2]. Since then, the number of cases identified in Italy rapidly increased, principally in the northern regions, with the major numbers of infections recorded in Lombardy. No health care system can be prepared to respond to such a rapid saturation of available beds in intensive care units and in the other departments of the hospitals. This fact translated into an exponential rise of the number of deaths and a reallocation of most of the available health care resources to treat COVID-19 patients, generating competition with other health care needs [3]. In particular, the Lombardy region created differentiated pathways for COVID-19 and non-COVID-19-related health services to better face the emergency [4].

Patients with cancer may be a vulnerable population during a pandemic. In fact, they are usually immunosuppressed because of their illness, treatment-related side effects, and nutritional deficiencies. In addition, they have an increased risk of developing opportunistic infections and severe complications related to these conditions [5].

Few data are available regarding the prevalence of COVID-19 infection in patients with a cancer history or in active oncological treatment [6]. A retrospective multicenter cohort study led by the Wuhan Union Hospital described an incidence of 25% of severe COVID-19 pneumonia in patients with cancer with a death rate of about 20%; in general, the case-fatality rate reported for cancer patients with COVID-19 was 18% [7]. In particular, male sex and having received chemotherapy within the four weeks before the onset of symptoms were identified as risk factors for death in patients with cancer and concomitant COVID-19. Among the various case series, the proportion of patients with COVID-19 and cancer varied between 0.5% and 6% [8]. Based on current evidence, patients with cancer might have a higher risk of acquiring SARS-CoV-2 infections and might have a higher risk of death from COVID-19 than those without cancer. Moreover, another recent study reported that cancer patients are at greater risk of developing severe complications compared to patients without cancer (39% versus 8%) [9].

Despite COVID-19 representing a serious threat for public health, cancer still remains one of the main causes of death. Postponing or modifying treatment schedule may lead to worse outcome, with a well-described impact on clinical outcomes. In addition, cancer patients may have a higher risk of infections because of the frequent access to the hospital. For this reason, oncology scientific societies have quickly released guidelines on cancer care during the pandemic, suggesting telemedicine services, reducing medical evaluations, switching to subcutaneous or oral therapies when possible, and evaluating the benefit of each treatment [10]. In particular, a detailed guideline document was published on 13 March by the Italian Association of Medical Oncology (AIOM) [11]. On 20 March, the National Comprehensive Cancer Network (NCCN) published recommendations for the management of cancer patients in endemic areas [12], followed by the European Society of Medical Oncology (ESMO) on 21 March [13].

Starting from these premises, the aim of this study was to investigate the management of cancer patients and the oncological treatments during the COVID-19 outbreak and to describe the containment measures performed in our outpatient clinic at Pavia. A comparison with the same period of the four previous years (2019, 2018, 2017, and 2016) was also performed.

## 2. Results

**Figure 1:**
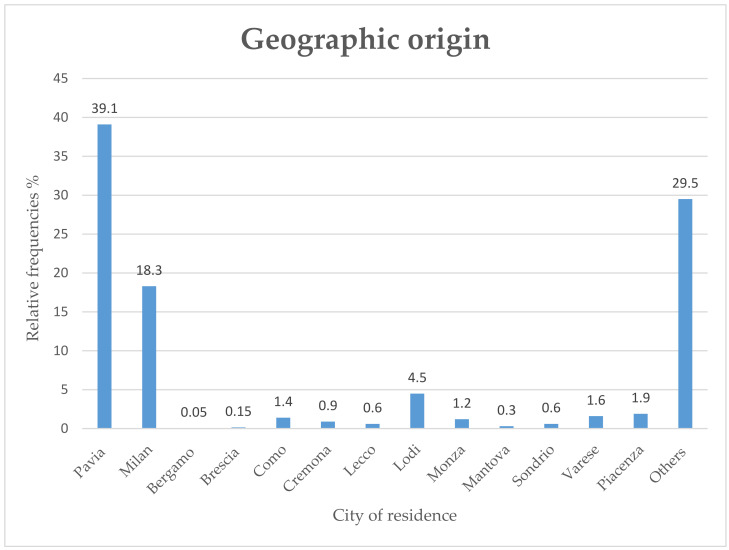
The image shows the distribution of the geographical area of the patients’ accessing to hospital in 2020.

A total of 469 patients accessed our division for cancer therapy during the lockdown period compared with 636 patients in the same period in 2019, 612 patients in 2018, 591 in 2017, and 604 in the year 2016. In 2020, the patients’ median age was 69 years (range 34–90), and 32% were male, whereas 68% were female. In the majority of cases, they came from Lombardy (68,7%) and in particular from Pavia and its province (39,1%), Milan and its province (18,3%), and Lodi and its province (4,5%) (Figure 1).

**Figure 2:**
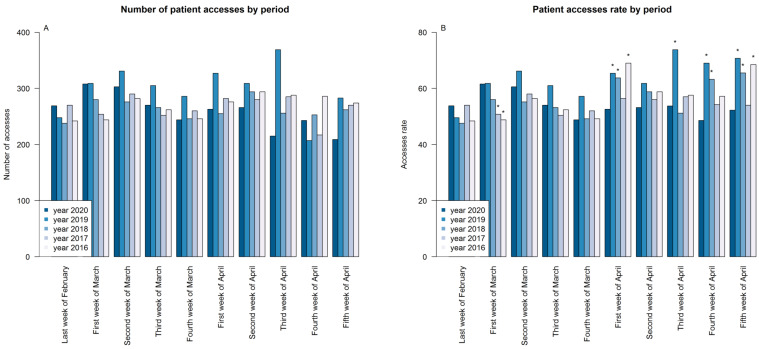
Absolute frequency of patient accesses (**A**) and patient accesses rate (**B**) by period. * Indicates statistically significant differences between access rates during 2020 and the considered year.

Concerning the primary objective, we recorded 2590 occasions of access during the COVID-19 pandemic compared to 2974 in the same period in 2019, 2626 in 2018, 2660 in 2017, and 2694 in 2016 (Table 1), and a statistically significant difference between the access rate during 2020 and 2019 was evident (53.96 versus 63.28, *p* < 0.001), but only a milder difference when comparing 2020 with 2018, 2017, and 2016 (*p* > 0.05). Performing an analysis by week, it can be noticed that during the first four weeks, the number of times accessed was similar between 2020 and the other years considered, declining progressively from the first week of April. Starting from this week, the rate of access during 2020 was generally lower compared to other years, in particular in comparison to 2019 and 2018 (Figure 2A,B, *p* < 0.05, Table 1).

During the pandemic, 72 patients started cancer treatment compared to 90 in 2019, 84 in 2018, 85 in 2017, and 88 in 2016.

**Figure 3:**
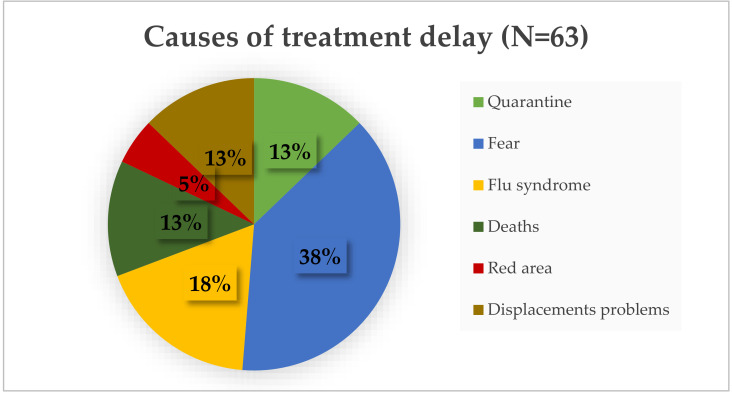
The image shows the COVID-related causes of treatment delay in 2020.

As a secondary objective, 63 patients had their treatment delayed due to COVID-19-related reasons (Figure 3). The median age of these patients was 70 years old; the majority had metastatic breast cancer (62%) and came from a city outside Pavia and its province (66%).

The fear of contracting the infection represented the most important cause of treatment delay (38%), followed by flu-like syndrome (18% of patients). Other reasons of delay were: quarantine (13%), deaths (13%), living in red areas (5%), and displacement problems due to closure of the region and province borders (13%). In the 2020 pandemic, 32 patients delayed treatment due to toxicity (20 cases) or worsening of clinical conditions (12 cases) compared to 45 patients in 2019 (25 for toxicity and 20 for worsening of clinical conditions), 39 in 2018 (22 for toxicity and 17 for worsening of clinical conditions), 41 (21 for toxicity and 20 for worsening of clinical conditions), and 35 in 2016 (18 for toxicity and 17 for worsening of clinical conditions). Only seven patients delayed/stopped active oncological treatments for ascertained COVID-19. The characteristics of patients who had COVID-19 are reported in Table 2.

The median age of these patients was 67 years old (range 40–81); two patients were male and five patients were female. All but one were affected by metastatic cancer for which they were receiving active treatments. Patients had a median of three comorbidities including cardiovascular and ischemic diseases; four patients died because of COVID-19, one patient was cured at home, one patient was hospitalized and cured, and one patient died from cancer disease progression with concomitant COVID-19, however, this did not represent the cause of death.

Moreover, during the lockdown period, 211 radiological exams for disease re-staging (computed tomography scans, positron emission tomography (PET) with 18-fluorodeoxyglucose or ultrasounds) were performed in patients visiting our cancer clinic for active treatments as compared with 360 exams in the same period in 2019, 335 exams in 2018, 385 in 2017, and 390 in 2016, with a statistically significant difference between the number of exams performed in 2020 and the other years (*p* < 0.001).

**Figure 4:**
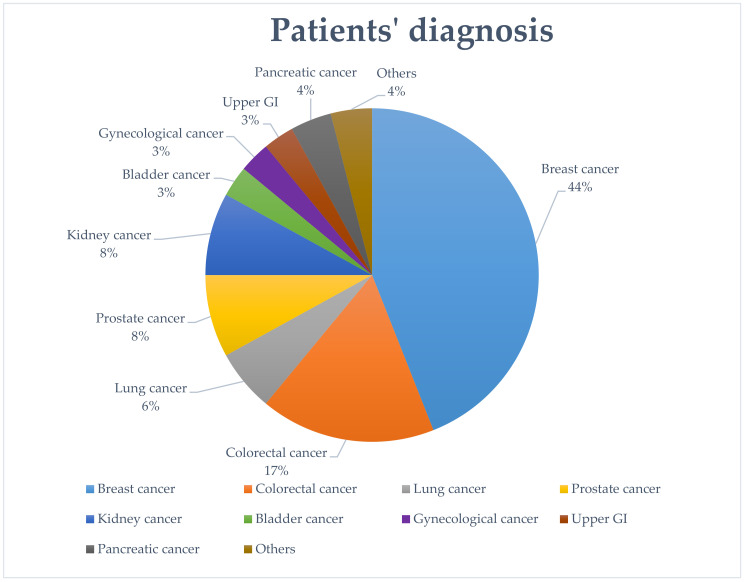
Image showing the diagnosis of patients accessing the hospital for cancer treatments in 2020.

We then analyzed the clinical characteristics of patients accessing our division in 2020, according to cancer type and treatment received (both treatment setting and type of drugs). Breast cancer was the most common diagnosis (44%), followed by colorectal cancer (17%), prostate cancer (8%), kidney cancer (8%), lung cancer (6%), pancreatic cancer (4%), gynecological (3%), bladder and upper gastrointestinal cancer (3%), and others (4%) (Figure 4).

**Figure 5:**
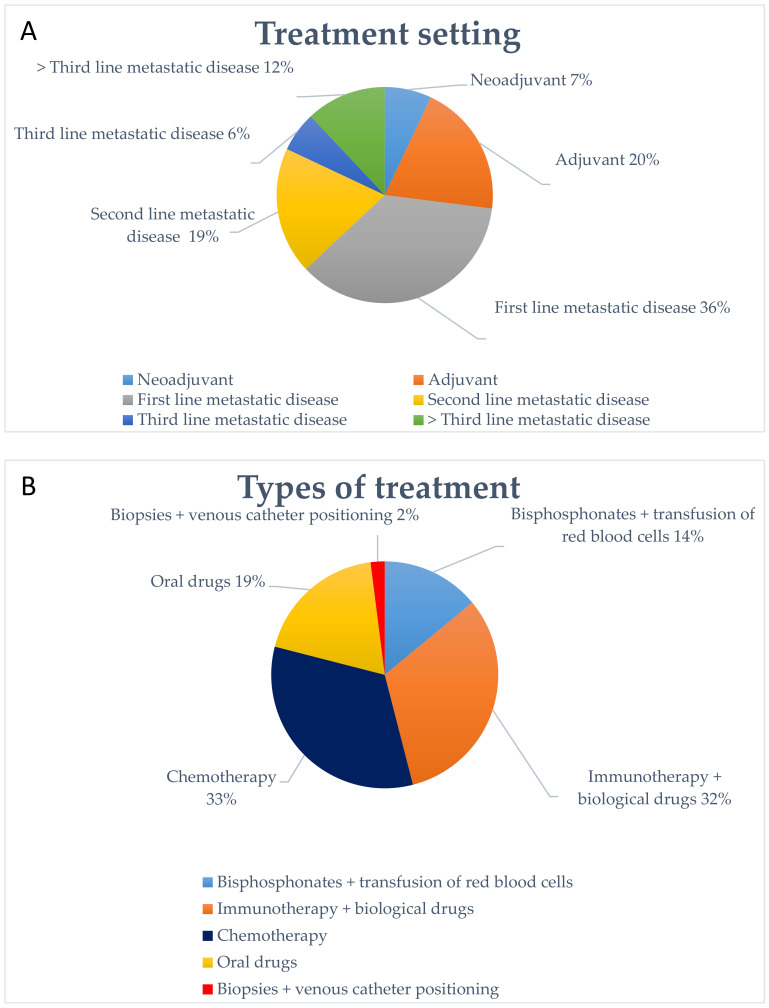
The image shows the type of treatment performed by patients (**A**) and patients’ setting of treatment in 2020 (**B**).

The majority of our patients were treated for a metastatic disease (73%), and in a minor percentage for an early stage cancer (33%). Among the treatments for advanced disease, the first line setting was the most represented and only a minority of patients accessed the hospital for advanced lines of treatment. In particular, 36% of patients received treatment as a first line setting, 19% as second line, 6% as third line, and 12% beyond the third line; moreover, 20% of patients received a treatment in adjuvant setting, while 7% in a neoadjuvant setting (Figure 5A).

We also analyzed the type of treatment performed. The majority of patients received an infusion therapy: chemotherapy (33.8%), immunotherapy, and/or biological drugs such as monoclonal antibodies (32%). The remaining patients received oral drugs (18.77%) or bisphosphonates and transfusion of red blood cells (13.75%) (Figure 5B). In 2020, 18 patients changed the treatment regimen from an intravenous infusion to an oral therapy to reduce the number of visits to the hospital. In addition, we performed complex diagnostic procedures such as liver biopsies or central venous catheter implantation in 2.28% of patients.

## 3. Discussion

Since the COVID-19 outbreak in February 2020, cancer hospitals in Lombardy and in Italy have reshaped the service delivery model and set priorities in cancer care. This is the first work that provides a quantitative assessment of cancer patient management during the pandemic. This study is particularly relevant if we consider that Lombardy was the Italian region with the highest incidence of COVID-19, with a total of 93,839 confirmed cases at the beginning of July 2020 (cumulative incidence of 932.74 × 100,000) and 5587 cases in the province of Pavia [14]. In addition, starting from the middle of March 2020, Lombardy and Pavia’s hospitals were completely dedicated to COVID-19 patients, thus reducing the possibility for patients to access the hospitals for visits, radiological, and blood exams. Only oncological services (for cancer patients in active treatment) and hemodialysis departments remained active in order to guarantee the continuation of life-saving treatments for these subgroups of patients.

Six papers described the management of cancer patients during the COVID-19 pandemic in Lombardy’s hospitals where specialized centers (hubs) with dedicated multidisciplinary “hub teams” were identified to treat oncologic patients [3,15,16,17,18,19]. In particular, these works discussed the hospital strategies to ensure the continuum of the best possible cancer care by estimating the individual patient’s risk of infection based on both the epidemiological considerations and individual clinical characteristics (in particular age and number of comorbidities). The key interventions that resulted aimed at: (i) reducing hospital visits and using telemedicine for follow-up visits; (ii) delaying medical tests and reserving radiological exams for patients with abnormal clinical findings; (iii) performing a telephone patient triage with checklist to investigate suspicious clinical symptoms or strict contacts during the previous three weeks with people having any symptoms of infection; (iv) creating a dedicated path for patients with symptoms or suspected contacts and the use of personal protective equipment (PPE) by the staff; and (v) modifying the schedule and route of administration for patients with ongoing treatment according to the expected benefit of maintaining the standard therapy. They also described the modality to manage surgical interventions and radiation therapy and to conduct clinical trials. A single investigation reported the reactions, attitudes, and countermeasures undertaken by medical oncology units facing the COVID-19 outbreak in southern Italy [20]. This study retrospectively obtained data from the time-related analysis of conversations via a phone messenger-based group chat between oncologists and patients, and concluded that the use of an instant-messaging system seemed to be a useful tool to share news and reactions between medical oncologists to rapidly implement necessary health measures and provide answers to most cancer patients’ needs. A recent paper investigated the impact of the COVID-19 pandemic on the attitudes and practice of Italian oncologists toward breast cancer and related research activities [21]. In particular, this work analyzed the results of a 29-question anonymous online survey that was sent by email to members of the Italian Association of Medical Oncology and the Italian Breast Cancer Study Group. The results describe a change in some oncologists’ attitude and practice as reasonable responses to the health care emergency (such as modifying weekly chemotherapy regimens to reduce patient hospital access or preferring home-taking oral therapies). However, some potentially alarming signals of under-treatment were observed and clinical research and scientific activities resulted in being reduced by 80.3% and 80.1%, respectively. Specific guidelines for the treatment of patients with gastrointestinal cancers [22] and older patients with cancer [23] were also published. In fact, older cancer patients may be denied supportive care because of their shorter life expectancy. The work provides special considerations to prevent the infection of older patients such as: a separate scheduling to protect them from being infected; the prompt activation of social services to ensure adequate medical supply, food and daily transportation to cancer centers; close monitoring through phone calls; shorter courses of radiotherapy; and home health care telemedicine to avoid hospital admission. The results of a national survey showed that containment measures for oncologic patients have been promptly implemented through the whole country, in particular, the use of protective devices, triage of patients accessing to the hospital, delay of non-urgent visits, and use of telemedicine [24]. A recent paper estimated the impact of delays in diagnosis on cancer survival outcomes in four tumors types in England and concluded that, due to the COVID-19 pandemic, an increase in the number of avoidable cancer deaths is expected in the UK [25]. In particular, a 7.9–9.6% increase in the number of deaths due to breast cancer up to five years after diagnosis, a 15.3–16.6% increase for colorectal cancer, a 4.8–5.3% increase for lung cancer, and a 5.8–6.0% increase for esophageal cancer is expected.

In our clinical practice, treatment decisions were initially provided on a case-by-case basis, aiming to guarantee the safety of patients and reducing the risk of progression due to treatment delays, actually anticipating the national guidelines. Since 24 February, we have implemented the recommendations and advices provided by scientific societies switching to oral treatments whenever possible and limiting the access of patients treated in advanced lines. Like in other centers, containment measures with limited hospital access and telephone triage were performed.

In this work, we described, from a quantitative point of view, the management and the results of the containment measured adopted by Istituti Clinici Scientifici (ICS) Maugeri and by the Oncology Unit with regard to the need of continuing the care of cancer patients reducing the risk of SARS-CoV-2 infection. The results of the comparison with the previous four years showed a reduction in the number of patients accessing the hospital for cancer therapies in 2020 compared with 2019, but not compared with 2018, 2017, and 2016. This fact may be due to the higher number of new cancer diagnoses in 2019 compared to in 2018, 2017, and 2016 and confirms that, during the pandemic, the hospital policy was able to establish an organization that allowed for the continuation of the oncology treatments. A statistically significant difference in the number of accesses was particularly evident between April 2020 and April 2019–2018. This fact cannot only be due to the treatment delays in 2020, but may depend on the closure of follow-up visits and radiologic services for routine exams and on the delay of the surgical intervention, reducing the number of possible new cancer diagnosis. In addition, a significant reduction in re-staging radiological exams in 2020 was evident when compared with the other years; this was due to the reduction in staff at the radiology unit and cancellation of programmed exams during the lockdown period. The treatments of patients accessing our clinic were continued in the presence of stable clinical conditions and no suspicions for disease progression. However, some disease progressions may not have been detected early, with an impact on clinical outcomes not clearly evaluable.

Concerning the geographical area, Pavia and its province was not one of the areas with the higher incidence of COVID-19, but Lombardy was the Italian region with the highest number of cases. The prompt adoption of containment measures, even before the national and international recommendations, may have contributed to containing the infection among our patients. In fact, we recorded only seven cases with COVID-19 in patients with a metastatic disease and several comorbidities. Moreover, cancer patients are usually strongly motivated to continue their treatments; for this reason, our patients immediately adopted the government’s indications to stay at home, respecting social distancing and individual restrictions, and they also reduced their contact with other people. On the other hand, cancer patients were worried that the immunosuppression and side effects of their treatment could lead to serious consequences in the case of COVID-19 and that a modification in their treatment plan could cause worse outcomes. In our study, a high percentage of patients were scared of going to the hospital because of the risk of infection. The psychological aspect of cancer patients in active treatment during COVID-19 is undoubtedly interesting and it can be assumed that in cancer patients, unlike the general population, the habitual coexistence of factors such as a severe disease, the presence of disabling symptoms and a condition of heavy dependence, together with the uncertainty of life control, would act as a protective element by minimizing or neutralizing the impact of the coronavirus in their individual scenario [26,27,28].

The study has some limitations: the monocentric and retrospective nature; SARVS-CoV-2 swabs were not performed as a screening measure in patients undergoing active cancer treatment so asymptomatic patients were not identified; the study lacked the capability to predict the impact of the delays of new cancer diagnosis, treatments, and instrumental disease re-staging on cancer prognosis.

## 4. Patients and Methods

### 4.1. Study Setting and Design

This was a retrospective, observational study in which, using our electronic databases, we evaluated the number of patients visiting the hospital for anticancer drug infusion and radiological exams from 24 February 2020 to 30 April 2020, taking into account for each patient:

- diagnosis;
- city of residence;
- setting of treatment and type of therapy performed;
- reason for a therapy delay.

The research was conducted according to the principles of the Declaration of Helsinki and was approved by the Local Institutional Ethical Committee (ICS Maugeri IRCCS Pavia Ethic Committee; approval number 2445). All patients involved in this study signed an authorization form for the use of their data.

### 4.2. Primary and Secondary Objectives and Endpoints

The primary objective of this study was to evaluate the performance of the Oncology Unit during the COVID-19 pandemic using an endpoint represented by the number of accesses for cancer therapy and start of new patients’ treatment in 2020, compared with the same period for the four previous years (starting from the last week of February until the end of April, for a total of 10 weeks considered for every year).

As secondary objectives, we aimed to:

- evaluate the impact of the pandemic on cancer care by assessing the number of therapeutic delays with the relative cause in 2020 compared to the four previous years;
- describe the therapeutic-diagnostic course of the patients through the number of treatment-related delays and the number of radiological exams carried out, compared to the same period of the four previous years;
- describe the setting of treatment (adjuvant/neoadjuvant/metastatic) and type of treatment performed in 2020;
- evaluate the grade of adherence to the recent national and international guidelines assessing the number of patients treated in first or subsequent lines and the switch to oral regimens.

### 4.3. Containment Measures

The Unit of Medical Oncology of IRCCS-ICS Maugeri is located in Pavia, Lombardy, and, like other areas of northern Italy, had to face the spread of COVID-19 with the necessity to ensure cancer care. Starting from the middle of March 2020, the majority of the departments of ICS Maugeri were converted into COVID-19 wards; surgeries and radiological services were limited to urgent procedures. Only the hemodialysis unit and cancer clinic continued their daily activity regularly.

A protocol of proactive management of cancer patients based on telephone triage and restricted access to the hospital was implemented, allowing the continuation of chemotherapies, biological treatments, targeted therapies, and immunotherapies. In particular, starting from 24 February, 2020:

- caregivers were not admitted to the hospital except in the case of non-self-sufficient outpatients;
- patients were asked to wear masks and gloves;
- outside the hospital, body temperature was measured and questionnaires asking for “flu-like” symptoms and possible COVID-19 contacts were performed;
- the day before the visit, a telephone triage was done in order to discuss with the patients their clinical conditions and possible symptoms related to COVID-19 including cough, sore throat, fever, dyspnea, myalgia, diarrhea, nausea/vomiting, anosmia, and dysgeusia;
- the day before the treatment administration, patients performed blood exams in separate hospital rooms to avoid crowding and allow an adequate organization of treatment infusions;
- patients that complained of suspicious symptoms or had contact with COVID-19 positive people were asked to call their doctor of general medicine for clinical evaluation or the designed number to seek appropriate medical support and not access the hospital for therapy;
- on the day of treatment administration, patients received an additional clinical triage at the hospital admission, with the evaluation of respiratory tract symptoms, possible contact with a COVID-19 positive person, and fever check;
- all healthcare personnel started to use PPE including wear masks, caps, disposable overalls, and gloves for the whole working day.

The staff of the Operative Unit of Oncology was not reduced in number and was totally dedicated to cancer patient care and not to COVID-19 patients. The usual number of 14 nurses and 10 physicians continued working; one nurse became infected, likely in the context of a familial cluster. ICS Maugeri created separated and independent access and paths for COVID-19 positive and negative patients with periodic environment sanitization.

Patient evaluation of the risk/benefit ratio for delaying anticancer treatment was based on several factors including:

- disease stage and histology;
- age;
- ECOG performance status;
- patient frailty and comorbidities;
- recent onset of tract respiratory symptoms or fever.

In frail patients with toxicities from the ongoing treatments or with a rapid worsening of clinical conditions, the treatment was delayed or definitively stopped.

In the case of oral treatments, in selected cases, drugs were supplied for longer periods (2–3 cycles) to reduce hospital access and patients were managed remotely by phone/email to evaluate possible adverse events, symptoms, and blood exams.

The routine follow-up visits were converted to telemedicine or postponed.

### 4.4. Statistical Methods

Continuous and discrete numeric variable distributions are described by the median and minimum–maximum range. Categorical variables distributions are described by absolute and relative (%) frequencies. The distribution of hospital accesses by categories is described by absolute frequencies and access rate by period (number of hospital accesses/total number of days considered). Access rates were compared between years by computing the access rate ratios or ARR (ratio between the access rate during 2020 and each year) and corresponding 95% confidence interval (95% CI). ARR values < 1 indicate that the access rate was lower during 2020 compared to the reference year, ARR values > 1 indicate that the access rate was higher during 2020 compared to the reference year, ARR values = 1 indicate that the access rate was unchanged between 2020 and the reference year. The *p*-value expresses the probability of obtaining an ARR estimate as discordant or more discordant with respect to the observed estimate, assuming that the null hypothesis is true (H0: ARR equals to 1, HA: ARR does not equal 1). The significance level was set to 0.05. Statistical analyses were performed by the R statistical software version 2.6.1 (www.r-project.org). ARR, corresponding to 95% CI and *p*-value were obtained by the *rateratio* function implemented in the *fmsb* package.

## 5. Conclusions

This study investigated the management of cancer patients during the COVID-19 outbreak in a Lombardy hospital and presented a report of the effects of the actions taken. A comparison with the same period of the four previous years (2019, 2018, 2017, and 2016) was also performed. The results of the analysis showed a slight reduction in patient visits for therapy in 2020, but this difference was significant only compared with 2019, but not with the other years. This fact may lead to the conclusion that the low incidence of COVID-19 among our cancer patients along with the hospital policy to control infection enabled the safe cancer treatment and continuum of care in most patients, while a small fraction of patients experienced a therapeutic delay due to patient-related reasons. The hospital system provided an acceptable continuum of care by minimizing the risks derived from treatment delays/modifications and in-hospital infections.

On the other hand, a statistically significant reduction in radiological exams as compared with the previous four years was recorded. The entity of the delay of new cancer diagnosis and the impact on the prognosis of cancer patients is not immediately evaluable, but it can represent a serious problem in the immediate future.

## Figures and Tables

**Table 1:** Number of patient accesses and access rates for 2020, 2019, 2018, 2017, and 2016 in each considered period.

Year	Last Week of February	First Week of March	Second Week of March	Third Week of March	Fourth Week of March	First Week of April	Second Week of April	Third Week of April	Fourth Week of April	Fifth Week of April	Total
2020	269/5 (53.8)	308/5 (61.6)	303/5 (60.6)	270/5 (54)	244/5 (48.8)	263/5 (52.6)	266/5 (53.2)	215/4 (53.75)	243/5 (48.6)	209/4 (52.25)	2590/48 (53.96)
2019	248/5 (49.6)	309/5 (61.8)	331/5 (66.2)	305/5 (61)	286/5 (57.2)	**327/5 (65.4)**	309/5 (61.8)	**369/5 (73.8)**	**207/3 (69)**	**283/4 (70.75)**	**2974/47 (63.28)**
	ARR = 1.08	ARR = 1	ARR = 0.92	ARR = 0.89	ARR = 0.85	**ARR = 0.8**	ARR = 0.86	**ARR = 0.73**	**ARR = 0.7**	**ARR = 0.74**	**ARR = 0.85**
	CI = 0.91–1.29	CI = 0.85–1.17	CI = 0.78–1.07	CI = 0.75–1.04	CI = 0.72–1.01	**CI = 0.68–0.95**	CI = 0.73–1.01	**CI = 0.62–0.86**	**CI = 0.59–0.85**	**CI = 0.62–0.88**	**CI = 0.81–0.9**
	*p* = 0.356	*p* = 0.968	*p* = 0.266	*p* = 0.144	*p* = 0.068	*p* = 0.008*	*p* = 0.073	*p* < 0.001 *	*p* < 0.001 *	*p* < 0.001 *	*p* < 0.001 *
2018	238/5 (47.6)	280/5 (56)	276/5 (55.2)	266/5 (53.2)	246/5 (49.2)	**255/4 (63.75)**	294/5 (58.8)	256/5 (51.2)	**253/4 (63.25)**	**262/4 (65.5)**	2626/47 (55.87)
	ARR = 1.13	ARR = 1.1	ARR = 1.1	ARR = 1.02	ARR = 0.99	**ARR = 0.83**	ARR = 0.9	ARR = 1.05	**ARR = 0.77**	**ARR = 0.8**	ARR = 0.97
	CI = 0.95–1.35	CI = 0.94–1.29	CI = 0.93–1.29	CI = 0.86–1.2	CI = 0.83–1.18	**CI = 0.69–0.98**	CI = 0.77–1.07	CI = 0.88–1.26	**CI = 0.64–0.92**	**CI = 0.67–0.96**	CI = 0.91–1.02
	*p* = 0.169	*p* = 0.248	*p* = 0.262	*p* = 0.863	*p* = 0.928	*p* = 0.028 *	*p* = 0.237	*p* = 0.599	*p* = 0.003 *	*p* = 0.015 *	*p* = 0.208
2017	270/5 (54)	**254/5 (50.8)**	290/5 (58)	252/5 (50.4)	260/5 (52)	282/5 (56.4)	280/5 (56)	285/5 (57)	217/4 (54.25)	270/5 (54)	2660/49 (54.29)
	ARR = 1	**ARR = 1.21**	ARR = 1.04	ARR = 1.07	ARR = 0.94	ARR = 0.93	ARR = 0.95	ARR = 0.94	ARR = 0.9	ARR = 0.97	ARR = 0.99
	CI = 0.84–1.18	**CI = 1.03–1.43**	CI = 0.89–1.23	CI = 0.9–1.27	CI = 0.79–1.12	CI = 0.79–1.1	CI = 0.8–1.12	CI = 0.79–1.13	CI = 0.75–1.08	CI = 0.81–1.16	CI = 0.94–1.05
	*p* = 0.966	*p* = 0.023 *	*p* = 0.593	*p* = 0.431	*p* = 0.476	*p* = 0.416	*p* = 0.549	*p* = 0.516	*p* = 0.239	*p* = 0.721	*p* = 0.827
2016	242/5 (48.4)	**244/5 (48.8)**	282/5 (56.4)	262/5 (52.4)	246/5 (49.2)	**276/4 (69)**	294/5 (58.8)	288/5 (57.6)	286/5 (57.2)	**274/4 (68.5)**	2694/48 (56.12)
	ARR = 1.11	**ARR = 1.26**	ARR = 1.07	ARR = 1.03	ARR = 0.99	**ARR = 0.76**	ARR = 0.9	ARR = 0.93	ARR = 0.85	**ARR = 0.76**	ARR = 0.96
	CI = 0.93–1.32	**CI = 1.07–1.49**	CI = 0.91–1.26	CI = 0.87–1.22	CI = 0.83–1.18	**CI = 0.64–0.90**	CI = 0.77–1.07	CI = 0.78–1.11	CI = 0.72–1.01	**CI = 0.64–0.91**	CI = 0.91–1.01
	*p* = 0.232	*p* = 0.006 *	*p* = 0.385	*p* = 0.729	*p* = 0.928	*p* = 0.002 *	*p* = 0.237	*p* = 0.443	*p* = 0.062	*p* = 0.003 *	*p* = 0.153

Each cell of the table reports: the total number of access/number of days considered (access rate), ARR = access rate ratio (ratio between the access rate during 2020 and each year), CI = ARR 95% Confidence Interval; *p* = *p*-value. As an example, during the first week of April 2020, a total number of 263 accesses were observed over five days (access rate = 52.6) while during the first week of April 2019, a total number of 327 accesses were observed over five days (access rate = 65.4). The ARR was 0.8 (52.6/65.4) with a confidence interval corresponding to 0.68–0.95, the access rate was significantly different between the two years (*p* = 0.008). * and bold numbers indicate statistically significant differences between access rates during 2020 and the considered year.

**Table 2:** Characteristics of patients with COVID-19.

Patient N	1	2	3	4	5	6	7
**Gender**	M	F	M	F	F	F	F
**Age (year)**	60	72	78	81	79	60	40
**ECOG PS**	1	2	2	1	2	1	3
**Cancer type**	Colorectal	Gastric	Prostate	Breast	Renal cell carcinoma	Neuroendocrine pancreatic tumor	Breast
**Stage**	Metastatic	Metastatic	Metastatic	Early	Metastatic	Metastatic	Metastatic
**Metastatic site**	Liver, lymph node, lung	Liver, lymph nodes, lung	Bone, liver	//	Lung, lymph node	Liver	Liver, lymph nodes,
**Setting of treatment**	First line	First line	Second line	Adjuvant	Second line	Second line	Fourth line
**Active treatment**	Oxaliplatin Fluorouracil Leucovorin	Cisplatin, Capecitabine, Trastuzumab	Enzalutamide Denosumab	Paclitaxel	Nivolumab	Everolimus	Exemestane plus everolimus
**Comorbidities Number**	4	4	4	2	2	2	0
**Type of comorbidities**	Blood hypertension, cardiac and renal failure, rheumatoid arthritis	Blood hypertension, hypothyroidism, HCV-related liver disease, heart transplant for dilated cardiomyopathy	Acute myocardial ischemia, chronic obstructive pulmonary disease, dyslipidemia, diabetes mellitus	Blood hypertension, osteoporosis	Blood hypertension, cerebral ischemia	Blood hypertension, diabetes mellitus	//
**Hospitalized (yes/no)**	Yes	Yes	Yes	Yes	Yes	No	No
**COVID-19** **status**	Died	Died	Died	Resolved	Died	Resolved	Died *

Abbreviations: ECOG PS: Eastern Cooperative Oncology Group Performance Status; F, female; HCV, hepatitis C virus; M, male; N, number * Died of disease progression and not from COVID-19.
